# Investigation of P-Type Cement Paste Production Through OH^−^ Ion Removal by Organic Acid Impregnation for Thermoelectric Energy Harvesting

**DOI:** 10.3390/ma18184229

**Published:** 2025-09-09

**Authors:** Hyun-Soo Lee, Jae-Suk Ryou, Hong-Gi Kim, Byeong-Hun Woo

**Affiliations:** 1 Civil & Environmental Engineering Department, Hanyang University Room 415, Jaesung Civil Engineering Building, 222 Wangsimni-ro, Seongdong Gu, Seoul 04763, Republic of Korea; lhw4005@hanyang.ac.kr (H.-S.L.); jsryou@hanyang.ac.kr (J.-S.R.); 2 Industry-University Cooperation Foundation, Daejin University, 1007, Hoguk-ro, Pocheon-si 11159, Republic of Korea; dmkg1404@daejin.ac.kr; 3 Department of Highway & Transportation Research, Korea Institute of Civil Engineering and Building Technologies, 283, Goyang-daero, Ilsanseo-gu, Goyang-si 10223, Republic of Korea

**Keywords:** cement paste, acid solution, acetic acid, citric acid, p-type, seebeck coefficient

## Abstract

Cement-based materials are reliable materials that guarantee high efficiency and high performance and are one of the important materials for the civil engineering industries. It is an era in which carbon neutrality and energy efficiency are emphasized, and electric energy production is currently being challenged even in cement paste. At this time, the most difficult part is the process of efficiently manufacturing a positive type of P-type cement paste. In this study, a P-type cement paste impregnated with an acidic solution to remove the OH^−^ ions was prepared, and the Seebeck coefficient was obtained to confirm its electrical production capacity. In the case of cement, specimens impregnated with acetic acid and impregnated with undiluted solution for 48 h showed the highest value at 1270 µV/K, and those impregnated with pure lemon juice showed the highest value at 1220 µV/K. It is estimated that the compressive strength of the cement paste impregnated with pure lemon juice is about 10–40% greater than that of the cement paste impregnated with acetic acid, so the condition for producing the optimal P-type cement paste is to impregnate the pure lemon juice in a solution diluted to 20% for 48 h. This study provides the possibility of manufacturing P-type cement paste with optimal performance and manufacturing electric production cement using the Peltier effect in the future.

## 1. Introduction

Cement and concrete are among the most widely used construction materials in civil engineering, and Ordinary Portland Cement (OPC), in particular, stands out as one of humanity’s most important consumed materials [1,2]. Cement is primarily utilized for its structural properties, forming the foundation of modern construction, ranging extensively from residential buildings to significant infrastructure projects such as bridges, dams, road pavement, and highways [3]. The primary constituents of cement paste—cement, water, and aggregates—interact through hydration to create durable and robust materials. However, the extensive scale of the cement industry carries substantial environmental costs, notably contributing approximately 7–8% of global CO_2_ emissions. This significant carbon footprint has heightened concerns regarding sustainability and climate change, prompting contemporary civil engineering research to increasingly emphasize eco-friendly and carbon-neutral cement-based solutions. Consequently, reducing the environmental impact of cement has become critical for achieving global climate objectives, motivating advancements in energy efficiency and developing energy harvesting capabilities within concrete structures [4,5,6].

One strategy to enhance concrete sustainability involves improving its energy efficiency through thermal energy storage. For instance, incorporating Phase Change Materials (PCMs) into cement composite allows these materials to absorb and subsequently release heat, effectively reducing heating and cooling demands in buildings [7]. PCMs can significantly enhance concrete’s thermal storage capacity by up to 14 times compared to traditional concrete [7]. Various studies have explored cement composite materials utilizing recycled glass aggregates [8], tetradecane [9], and recycled aggregates [10]. Nonetheless, the known disadvantage of PCM inclusion is its detrimental effect on mechanical properties [9,11]. PCM-impregnated concretes typically experience reductions in stiffness and compressive strength, substantially compromising structural stability. This limitation restricts the practical use of PCM-based energy-efficient concrete in structural load-bearing applications, necessitating alternative strategies that enhance energy efficiency without sacrificing mechanical performance [9].

An alternative promising approach is exploiting the thermoelectric effect within cement composites, directly converting temperature differentials into electricity. Unlike PCM systems, which store thermal energy passively, thermoelectric materials actively generate electrical power from temperature gradients [12]. Concrete structures naturally exhibit temperature gradients due to external solar heating and internal temperature differences, creating an opportunity to leverage concrete itself as a thermoelectric material capable of converting heat energy into electrical energy [12,13]. The Seebeck effect generates voltage when a temperature difference exists across a material, effectively transforming waste thermal energy into electricity. The conversion efficiency is characterized by the Seebeck coefficient (SC), defined as the voltage generated per unit temperature gradient. Recent research efforts have concentrated on enhancing the SC of cement-based materials through incorporating conductive or semiconductive additives, such as ZnO [14], reduced graphene oxide [13,15], and carbon nanotubes [16], yielding significant improvements in thermoelectric performance. These modified cement composites maintain structural integrity while producing electricity, effectively transforming concrete into a multifunctional structural material serving as its own thermoelectric generator, thereby potentially enabling self-powered buildings and infrastructure, contributing to overall energy efficiency, and reducing carbon footprints.

A critical challenge in developing thermoelectric cement paste involves achieving P-type (Positive-type) behavior, essential for creating effective thermoelectric circuits. OPC paste inherently demonstrates N-type thermoelectric behavior primarily due to its highly alkaline pore solution rich in OH^−^ ions, acting as charge carriers. Traditionally, P-type cement paste has been produced by doping cement with P-type conductive additives. For example, Agbaoye et al. [17] applied aluminum coatings onto cement hydration products, and Ji et al. [18] introduced MnO_2_ nanorods into cement composites to shift the SC. Although these approaches attained some P-type behavior, they exhibited notable drawbacks. The addition of metal oxides or nanomaterials typically involves complex pretreatment processes, poor dispersion within the cement composites [12], and significant economic drawbacks due to the high costs associated with CNTs and specialized semiconductors. Furthermore, materials such as bismuth-telluride alloys have been explored to enhance thermoelectric performance but raise cost, compatibility, and environmental concerns, underlining the necessity for simpler, cost-effective, and sustainable methods.

To address these limitations, this study investigates a simpler, economically viable method to induce P-type thermoelectric behavior in cement paste through acid impregnation treatments. The core concept involves chemically neutralizing or removing excess OH^−^ ions from cured cement using acidic solutions, thereby altering the internal electron donor balance and shifting behavior from N-type (Negative-type) toward P-type. Unlike traditional doping methods, acid impregnation employs readily available, cost-effective acid solutions without introducing new solid additives into the cement paste. By systematically varying the type and concentration of the acidic solution and impregnation duration, optimal conditions for producing P-type cement paste were identified. This research evaluates the effectiveness of acid impregnation in OPC paste under various conditions, demonstrating its potential as a practical, scalable method for producing P-type cement paste suitable for future thermoelectric energy harvesting applications.

## 2. Materials and Experimental Methods

### 2.1. Entire Experiment Process

**Figure 1:**
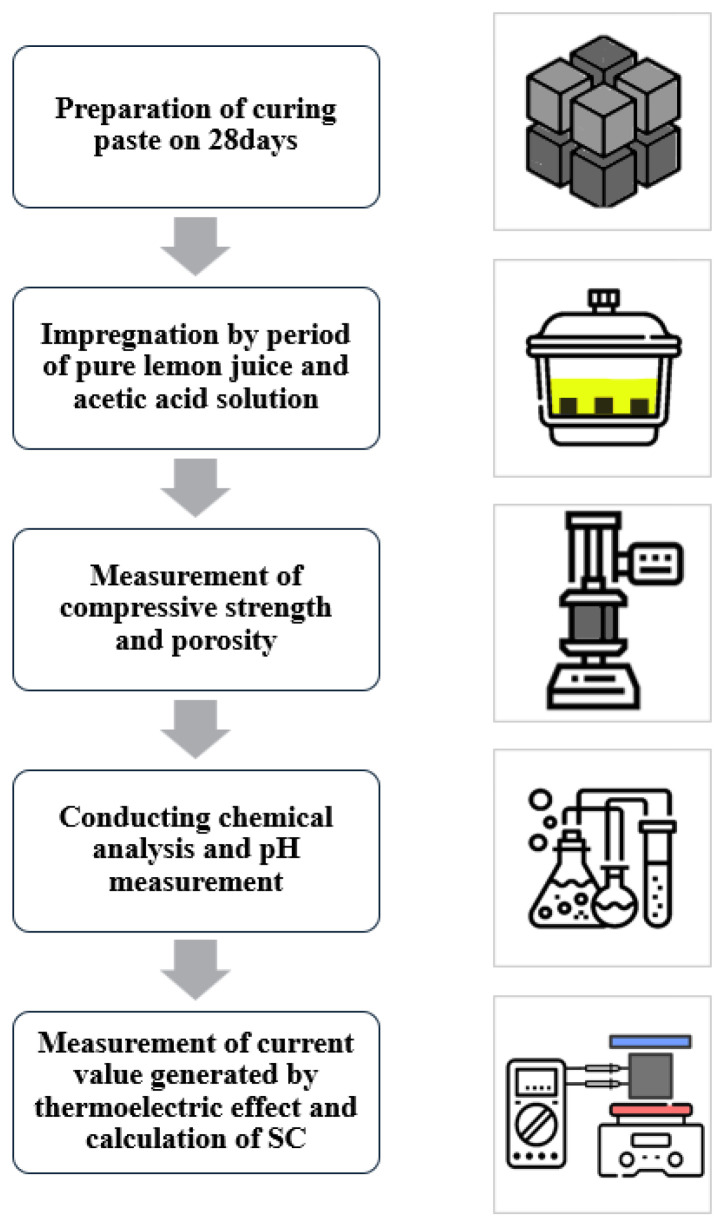
Schematic diagram of the entire experimental process.

Following Figure 1 is a schematic diagram of the progress and order of the experiment to be conducted in this study. After curing the cement paste prepared according to the mixing ratio for 28 days, each cement paste is impregnated with the acid solution for 1 day, 2 days, and 3 days. After that, compression strength tests are conducted to understand the mechanical properties of the finished test specimen, and XRD, DTS/TGA, SEM, porosity, and alkaline analyses are carried out for chemical characteristic analysis. Finally, the SC values are measured and analyzed to evaluate the test sample’s electrical production capacity.

### 2.2. Materials and Specimen Preparation

**Figure 2:**
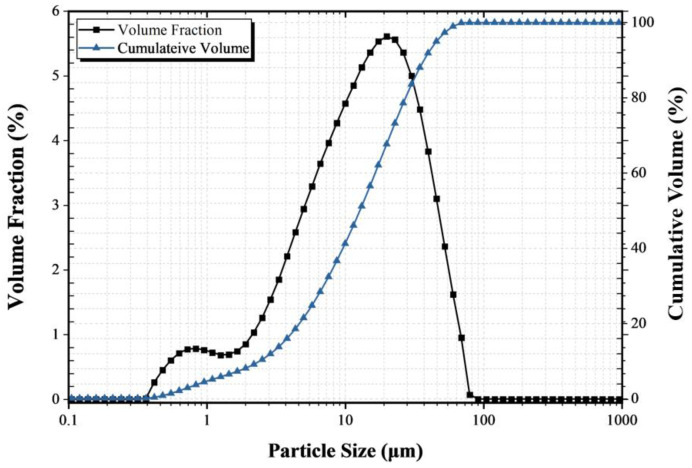
Particle size test results.

In this study, the cement used is OPC according to ASTM C150/C150M-18 [12,19]. Table 1 below contains the chemical and physical properties of OPC. Also, Figure 2 shows the particle size graph for the particle size test results of the OPC used in this experiment. Using the aforementioned OPC, cement paste with a water-cement ratio (W/C) of 40% was prepared into cubes with dimensions of 50 × 50 × 50 mm^3^ and cured for 28 days.

The cement paste was exposed to acidic solutions mixed by type using pure lemon juice and 100% acetic acid. Lemon juice was purchased from the market, containing 99.97% lemon juice concentrate and 0.03% antioxidant additive. Acetic acid is a corrosive organic acid characterized by a sharp odor, burning taste, and harmful fumes. It occurs in trace concentrations in seawater, brines, rain, and the fluids of many plants and animals. Table 2 below presents the physical properties of acetic acid [20].

Table 3 shows the results of the XRF test analysis to analyze the chemical characteristics of acetic acid and lemon juice used in the experiment. Five components, including trace amounts of C_6_H_10_O_5_ and Cl Fe, were measured in acetic acid, and a total of 10 components, including the same components as acetic acid and additionally Mg and P, were detected in lemon juice.

### 2.3. Impregnation Condition

Table 4 indicates the mixing ratios of acidic solutions for each proportion. The compositions are categorized as follows: cement paste not impregnated in an acidic solution (OPC), cement paste impregnated in a 100% acetic acid solution (A100), cement paste impregnated in 50% acetic acid and 50% water (A50), and cement paste impregnated in a solution of 20% acetic acid and 80% water (A20). Similarly, lemon juice, like acetic acid, was diluted with water at ratios of 100%, 50%, and 20%, labeled as L100, L50, and L20, respectively.

The acidic solutions prepared according to Table 4 were placed in desiccators, and the specimens were fully impregnated in them. Using a vacuum pump, the internal state of the desiccators was maintained under a vacuum, and each was cured for 1, 2, and 3 days, respectively. The test specimens were named according to the impregnation period and labeled 1, 2, and 3, respectively.

### 2.4. Strength and Chemical Property Test

#### 2.4.1. Compressive Strength Test

In this study, compressive strength tests were performed to evaluate the mechanical properties of the specimens impregnated in acidic solutions. Compressive strength tests were performed on OPC specimens not exposed to acidic solutions and specimens impregnated in acidic solutions for 24, 48, and 72 h after 28 days of curing. The compressive strength test utilized cubic specimens measuring 50 × 50 × 50mm^3^, following ASTM C109/C109M-21 standards [21].

#### 2.4.2. Porosity Test

**Figure 3:**
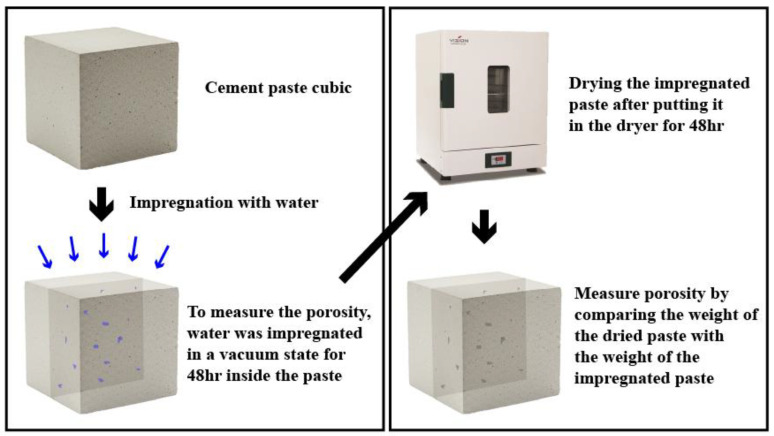
Process of porosity test.

A porosity test was conducted to examine the internal pores of the cement paste impregnated in an acidic solution. The internal pores of the cement paste were analyzed as illustrated in the schematic diagram in Figure 3.

The impregnated cement sample was placed in a desiccator with distilled water and maintained under vacuum conditions for 48 h. The weight was measured after the pores were filled with distilled water. Subsequently, the specimen was placed in a drying oven and completely dried at 80 °C for 48 h [22,23]. After all the distilled water inside the pores had evaporated, the weight of the pure cement paste was measured and calculated using the following Equation (1) [22,23]. $\rho_{w}$ is the density of water (kg/m^3^), $m_{s}$ is the weight (kg) of the cement paste saturated with distilled water, $m_{d}$ is the weight (kg) of the fully dried cement paste, and $V_{vol}$ is the volume (m^3^) of the cement paste.

(1) $$\emptyset =\frac{m_{a}-m_{d}}{\rho_{w}\cdot V_{vol}}$$

#### 2.4.3. X-Ray Diffraction (XRD) Test

X-ray diffraction (XRD) crystal analysis was conducted to confirm the presence of calcium hydroxide (CH) crystals within the specimens impregnated in acidic solutions. As a pretreatment for XRD analysis, each specimen was ground into a powder form and sieved through a #200 mesh sieve. Subsequently, XRD analysis was carried out over an angular range of 5 to 90 degrees. The tests were conducted following the ASTM E3294-22 standard [24].

#### 2.4.4. Differential Thermal Analysis (DTA)/Thermogravimetric Analysis (TGA) Test

Through differential thermal analysis (DTA) and thermal weight analysis (TGA), the weight change of the sample impregnated in the acidic solution was quantitatively analyzed according to temperature to determine the temperature stability and the ratio of volatile components. The tests were conducted following ASTM E1131-20 standards [25]. As a pretreatment for DTA/TGA analysis, each specimen was ground into a powder form and sieved through a #200 mesh sieve. Subsequently, DTA/TGA analysis was carried out with a maximum temperature of 1000 °C and a heating rate of 10 °C/min, using N_2_ gas.

#### 2.4.5. Scanning Electron Microscopy (SEM) Test

To observe the presence of CH crystals within the paste specimens, a scanning electron microscopy (SEM) test was carried out. SEM scanning followed the ASTM F1372-93 standard [26].

#### 2.4.6. Effective Alkaline Area (EAA) Test

**Figure 4:**
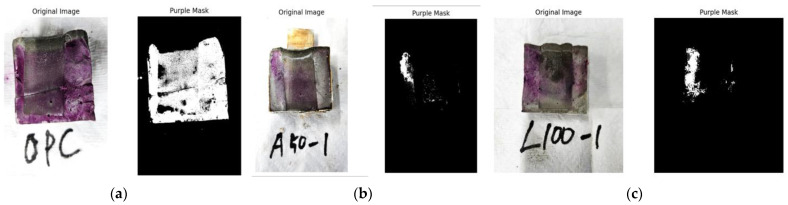
Examples of effective alkaline area extraction through image processing: (**a**) OPC; (**b**) A specimen; (**c**) L specimen.

An effective alkaline area (EAA) test was conducted to verify whether the OH^−^ groups inside the cement paste impregnated in the acidic solution were sufficiently removed. The EAA test was performed following ASTM C1910/C1910M-23. A solution with a concentration of 1% phenolphthalein is sprayed onto the cross-section of the cement paste to cause a colorless reaction when the pH of the cross-section is 9 or less, and a purple color change is observed when the pH is 9 or more. After the test, images of the cement paste specimens were processed as shown in Figure 4, extracting only the purple regions that reacted with phenolphthalein. Finally, the ratio of the purple region to the total cross-sectional area of the specimen was calculated to determine the effective alkaline area ratio.

#### 2.4.7. Electrical Resistivity Test

**Figure 5:**
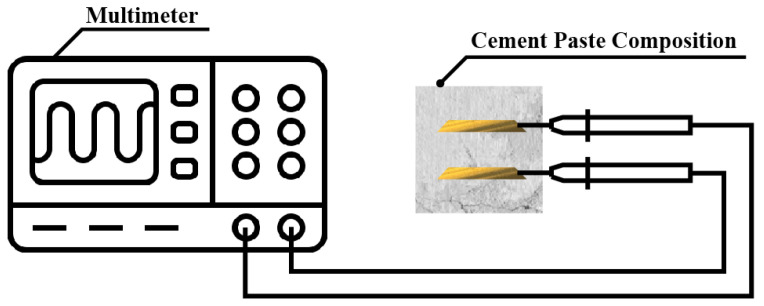
Electrical resistivity test.

To measure the electrical resistivity of each specimen, the negative and positive probes of the multimeter were connected to the embedded copper plate of the specimen. The resistance value is set to 1 s. Once, it was recorded in the 1800 s. The following Figure 5 is a schematic diagram of the electrical resistance test process.

#### 2.4.8. Measurement of Seebeck Coefficient Test

**Figure 6:**
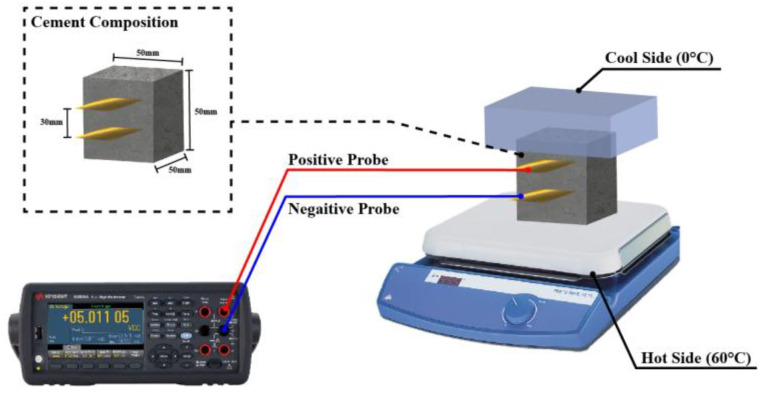
Measurement Seebeck coefficient process for hot plate using a multimeter.

In this study, the thermoelectric properties of the specimen were evaluated by measuring the SC. The specimen used in the test was 50 × 50 × 50 mm^3^, and 20 × 50 × 0.5 mm^3^ copper plates were installed at 30 mm intervals at the top and bottom of the specimen, as shown in Figure 6. Then, as shown in Figure 6, 50 × 50 × 1 mm^3^ copper plates were placed up and down to improve thermal transferring. A cooling system was installed at the top and a hot plate at the bottom to induce temperature changes in the ∆T. ∆T was set to 60 °C, indicating the best SC in the existing study [12]. The lower hot plate remained at 60 °C and the upper cooling system remained at 0 °C for 90 min. The copper plate near the hot plate was connected to the negative probe of the multimeter, and the copper plate on the cooling side was connected to the positive probe of the multimeter. The voltage generated from the specimen using a multimeter sensor was recorded every second, and the final SC was calculated by Equation (2).

(2) ∆V=S×∆T

where S is the SC (μV/K) value of the test object, and ∆T is the temperature difference provided to the test object and is fixed at 60 °C in this study. ∆V represents the thermoelectric voltage generated by the temperature difference provided to the cement paste.

## 3. Results and Discussion

### 3.1. Compressive Strength Results

**Figure 7:**
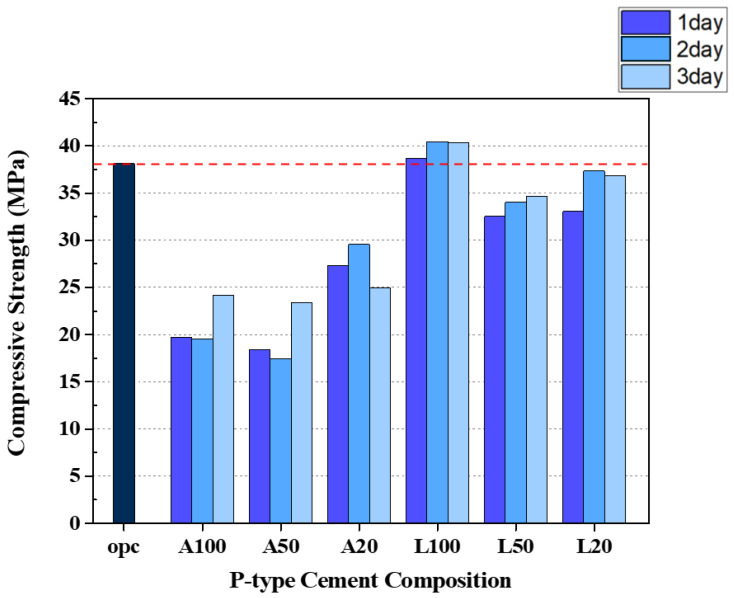
Compressive strength test result.

Figure 7 shows the results of the compressive strength measurements of each cement composition after impregnation in a solution for a different period. The compressive strength of OPC was measured at 38.14 MPa. In the case of acetic acid, the strength of the A50 specimen decreased over the impregnation period of one, two, and three days. Similarly, L50 specimens impregnated in lemon juice at a concentration of 50 percent showed the lowest strength in the cases of the L series. Overall, the compressive strength of cement paste impregnated in acetic acid was lower than that of cement paste impregnated in lemon juice. In particular, L100 samples (100% lemon juice) showed higher compressive strength than OPC overall impregnation periods (1 day, 2 days, 3 days).

**Figure 8:**
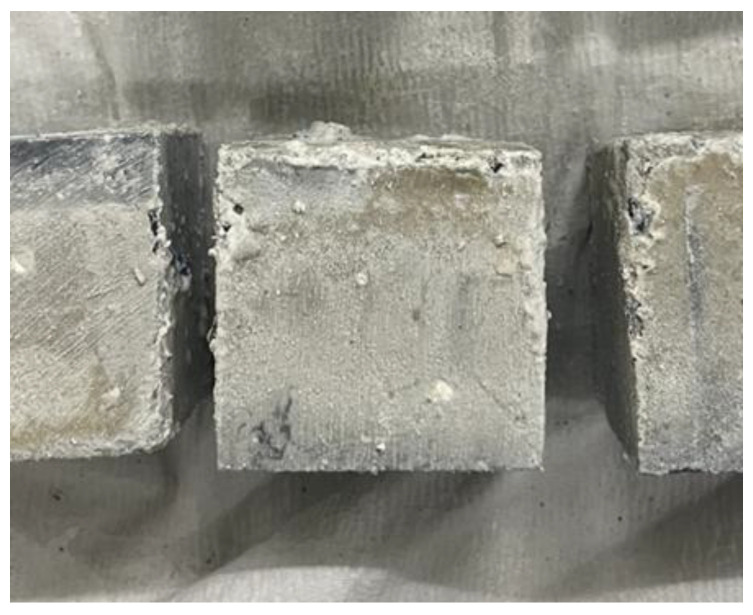
Calcium carbonate film formed on specimen surface.

This result is attributed to the reaction between citric acid (C_6_H_8_O_7_), which is the main component of lemon juice, and CH, which is the product of the cement paste hydration process. This reaction produces calcium carbonate (CaCO_3_) as described in Equations (3) and (4) [27,28]. The resulting calcium citrate (CaC_6_H_5_O_7_) [28] reacts with water and carbon dioxide (CO_2_) in the air to form CaCO_3_. Calcium carbonate, which is observed as a solid insoluble in water, is formed on the surface of the specimens like Figure 8.

(3) $$Ca{\left(OH\right)}_{2}+C_{6}H_{8}O_{7}\;\to \;CaC_{6}H_{5}O_{7}+2H_{2}O$$

(4) $$CaC_{6}H_{5}O_{7}+CO_{2}+H_{2}O\;\to \;CaCO_{3}+C_{6}H_{8}O_{7}$$

**Figure 9:**
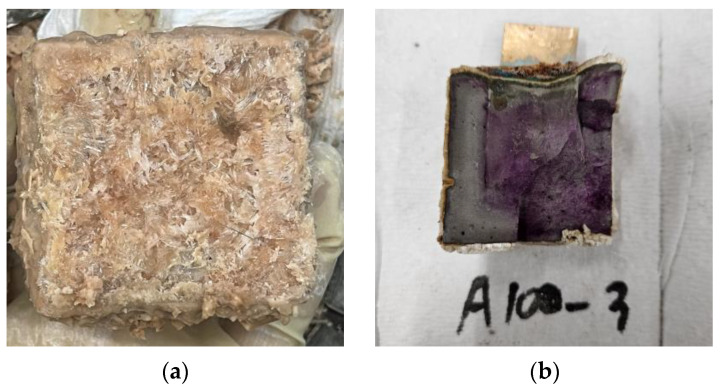
Changes in cement paste due to impregnation of acetic acid: (**a**) Calcium acetate film formed on concrete paste surface; (**b**) Cross section of cement paste impregnated with acetic acid.

In the cases of the A series specimens, the surface of the cement composition was significantly corroded after impregnation in the solution, and the crystal surface was observed as shown in Figure 9. This is due to the exposure of the hardened cement matrix to acetic acid, which produces calcium acetate [29,30,31] and forms a membrane on the surface of the A series specimens. The reaction equation is given by Equation (5). However, acetic acid tends to decalcify the hydrated cement phase of the hardened cement paste actively [29,30], which reduces the compressive strength compared to the relatively high acidity of lemon juice.

(5) $$Ca{\left(OH\right)}_{2}+2CH_{3}COOH\;\to \;Ca{\left(CH_{3}COO\right)}_{2}+2H_{2}O$$

### 3.2. Porosity Analysis Results

**Figure 10:**
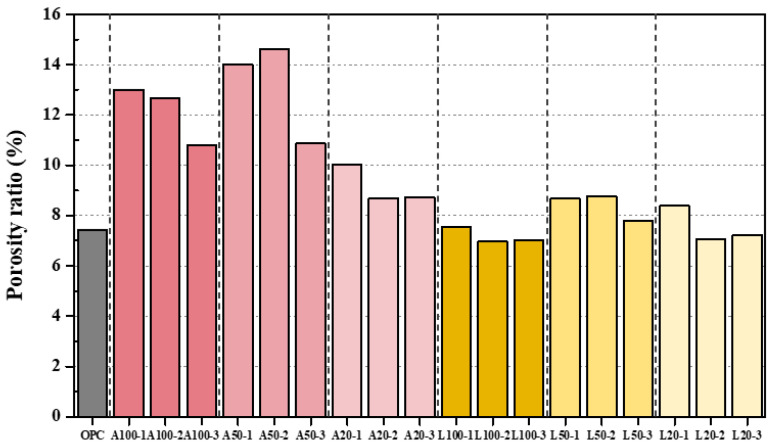
Porosity test result.

The following Figure 10 presents the porosity results of the cement paste impregnated in an acidic solution in graphical form. The porosity of OPC was measured at 7.4%. Among the A specimens, A20-2 exhibited the lowest porosity at 8.68%, while A50-2 showed the highest porosity at 14.4%, more than twice the porosity of OPC. In contrast, for the L specimens, L100-2 exhibited a porosity of 7.06%, which was 0.87% lower than that of OPC, while L50-2 recorded the highest porosity at 8.78%. The difference in porosity between the A and L specimens is closely related to the compressive strength results of part 3.1 [32]. Specifically, A50 exhibited the highest porosity in the A specimens, whereas A20 had the lowest porosity. This trend aligns with the compressive strength results, where A50 showed the lowest compressive strength and A20 exhibited the highest compressive strength. Similarly, for the L specimens, L50 showed the highest porosity, while L100 had the lowest porosity. In terms of compressive strength, L50 recorded the lowest value, whereas L100 exhibited the highest compressive strength.

In this experiment, porosity is a key factor in determining the mechanical strength of cementitious materials. In general, higher porosity corresponds to lower compressive strength, as described by classic gel-to-pore space ratio models [33]. An acid attack affects this relationship by altering the volume and pore structure of the gel (solid reaction product). When the acid dissolved CH and demineralized CSH, the volume of the cement paste decreased and the pore volume increased, which reduced the load-bearing cross-sectional area and consequently the compressive strength [34]. Essentially, the acid-induced porosity reduced the gel-to-pore space ratio (the ratio of the solid hydrated gel to the total (gel + pore) volume) and thus the strength [35]. A lower gel-to-pore space ratio is thought to result in a lower number of solid bonds per unit volume, which in turn reduces the strength of the material under compressive load [36,37]. This observed trend of compressive strength reduction is particularly pronounced in specimens impregnated with acetic acid. This can be attributed to the stronger acidity of acetic acid, which exerts a more significant impact on internal hydration products, specifically CH and CSH, compared to the relatively weaker citric acid [38]. In contrast, the formation of a CaCO_3_ film within the citric acid-impregnated L specimens contributes to enhanced compressive strength by sealing internal pores and increasing surface densification. Consequently, this mechanism effectively explains the difference in compressive strength observed between the A and L specimens, as shown in Figure 7.

### 3.3. XRD Analysis Results

**Figure 11:**
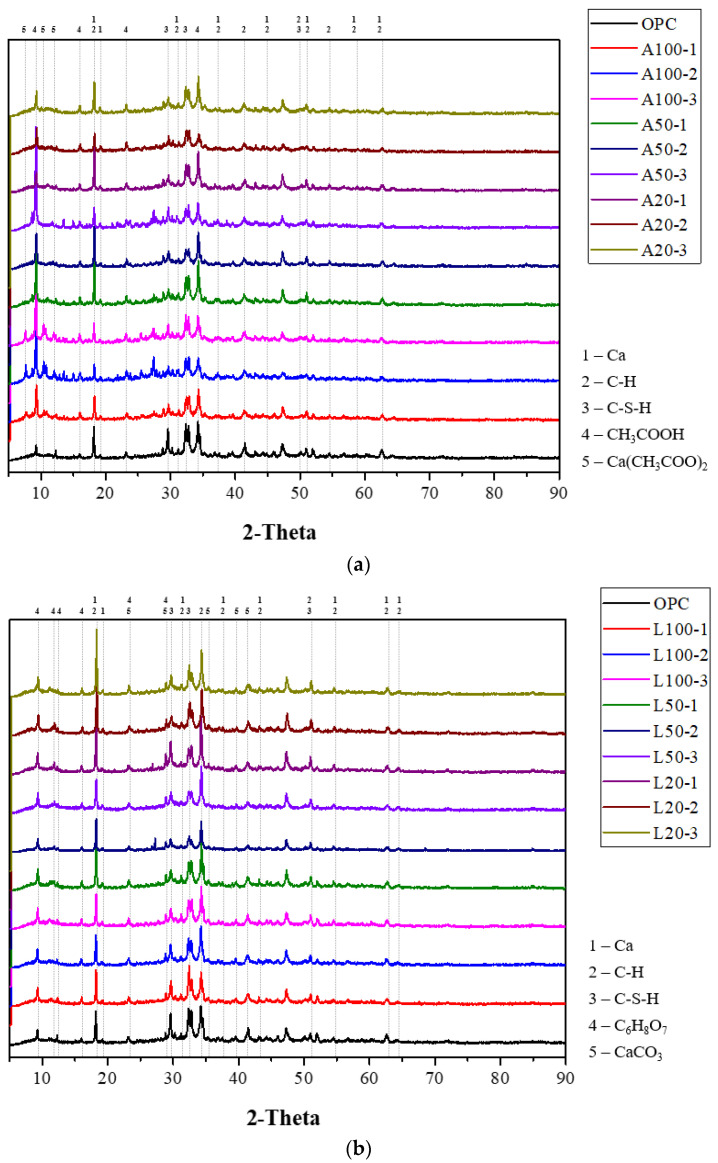
XRD analysis results: (**a**) A specimens and OPC; (**b**) L specimens and OPC.

Figure 11 shows the XRD analysis results for each specimen. Figure 11a shows the results of OPC, A100, A50, and A20 samples, and Figure 11b shows the results of OPC, L100, L50, and L20 samples. In both (a) and (b), the CH phases produced during the hydration process of cement appear at approximately 19°, 34°, 47°, 51°, and 54° [39,40,41], and the CSH phases appear at approximately 29°, 32°, and 50° [42]. Each of these phases has its own; they are labeled number 3 and 4.

CH produced by the cement hydration reaction was also observed at approximately 32°, 37°, 45°, 56°, 59°, and 66° [43,44,45,46] in Figure 11, shown as 1. For the A series specimens, A50 and A20 showed similar peaks to OPC, and A100 showed relatively low peaks at labeled number 1. Ca observed after removal of OH^−^ from Ca(OH)_2_ was observed at approximately 19°, 32°, 37°, 45°, 56°, 59°, and 66° [47], which were marked as number 2. Calcium acetate produced by exposure of hardened cement to acetic acid was observed at approximately 6°, 10°, 12°, and 14° [48], which were marked as number 6.

As for the L series specimens, the overall peak value labeled number 1 was lower than that of OPC. As a result, the peak of labeled number 2 corresponding to the removal of OH^−^ from CH was high, especially in L20 specimens compared to OPC. The peaks of calcium carbonate produced by the reaction of citric acid, the main component of cement and lemon juice, were observed at about 23°, 29°, 38°, 40°, and 43° [47,49,50], which were marked as number 6.

Acetic acid was observed at about 9°, 16°, and 23° [51] in graph (a), and citric acid, the main component of lemon juice, was observed at about 14°, 17°, 24°, and 27° [52,53,54] in graph (b). These were marked number 5 in both (a) and (b).

Through the results of the XRD experiment, the peak of Ca^2+^ ions produced by removing OH^−^ groups from the cement paste impregnated in the acidic solution was observed, and the products of Equations (3)–(5) were observed. Studies observing Ca^2+^ are rare, but similar trends are often observed in acid resistance studies [55,56]. Through this, it is considered that the production of the P-type in which the OH^−^ groups for the purpose of this experiment were removed was successful.

### 3.4. DTA/TGA Analysis Results

**Figure 12:**
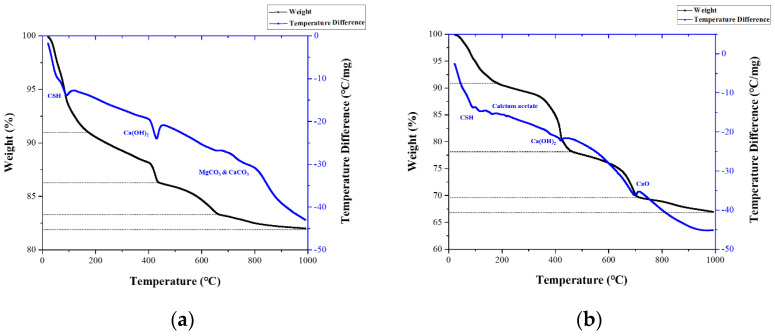

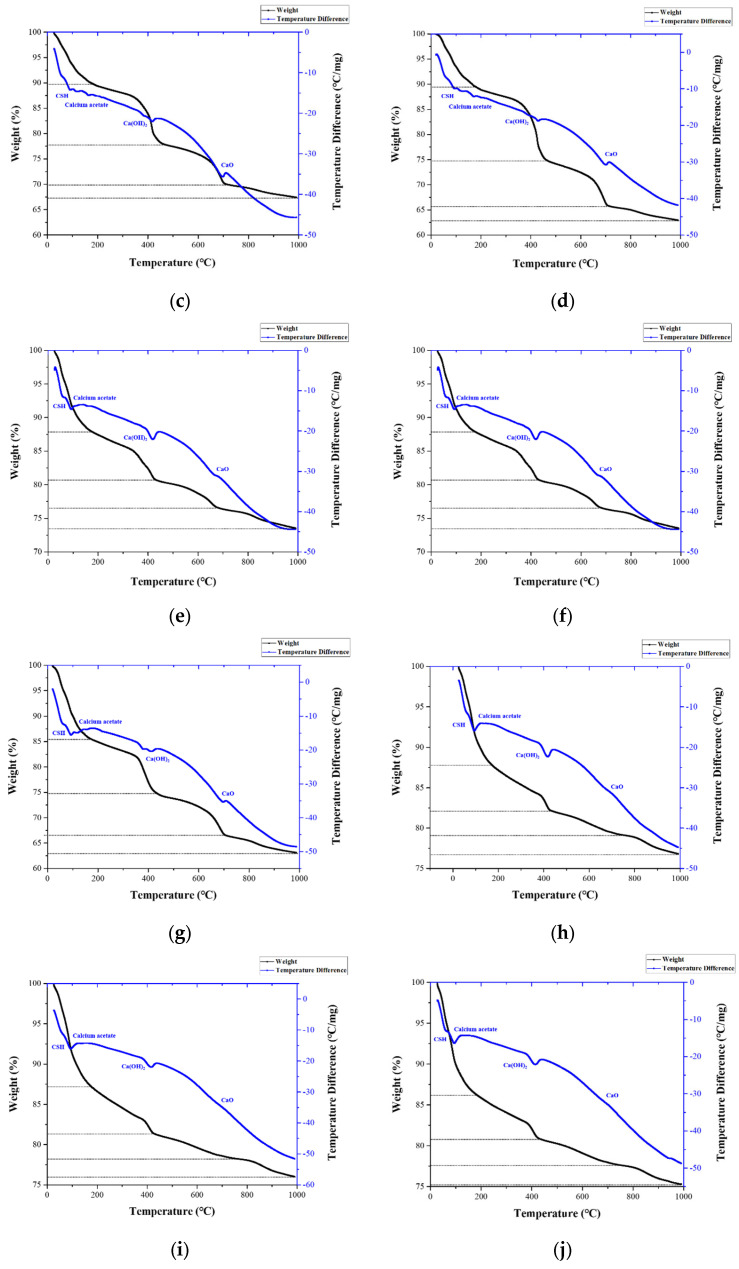

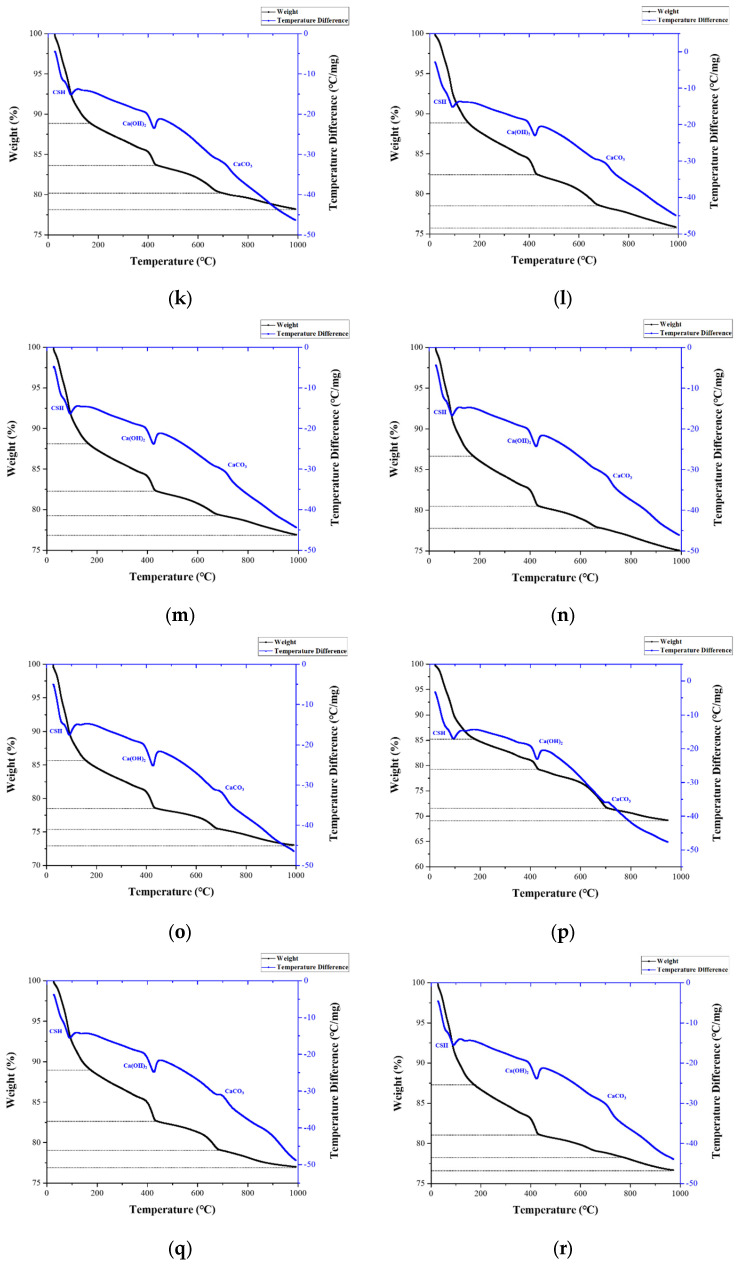

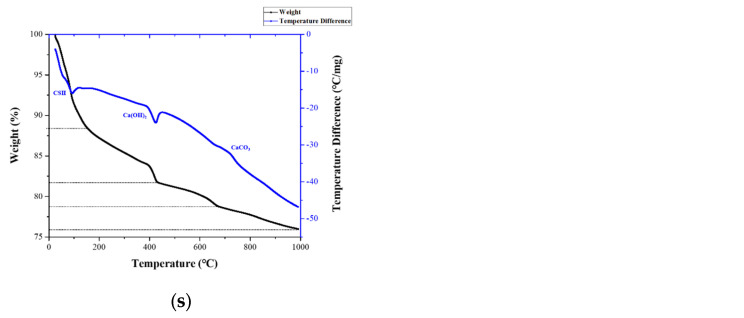
DTA/TGA analysis results: (**a**) OPC; (**b**) A100−1; (**c**) A100−2; (**d**) A100−3; (**e**) A50−1; (**f**) A50−2; (**g**) A50−3; (**h**) A20−1; (**i**) A20−2; (**j**) A20−3; (**k**) L100−1; (**l**) L100−2; (**m**) L100−3; (**n**) L50−1; (**o**) L50−2; (**p**) L50−3; (**q**) L20−1; (**r**) L20−2; (**s**) L20−3.

Figure 12 depicts the results of the DTA/TGA analysis for each specimen. The peaks of the TGA mass change and DTA graphs show the breakdown of bonds present in the sample at a given temperature. The same is true for OPC, A, and L cement specimens; the endothermic peaks between 100 °C and 200 °C in the DTA graph generally indicate the breakdown of the bonds of CSH produced during the cement hydration process [57,58,59]. Figure 12a In the case of OPC, MgCO_3_ and CaCO_3_ peaks appear at about 600–800 °C [60]. In addition, the endothermic peak of the DTA graph indicates decarboxylation of Ca(OH)_2_ between 400 °C and 600 °C [57,58,60]. Therefore, the TGA graph shows the mass loss in the same temperature range where endothermic peaks of CSH, CH, and Ca(OH)_2_ appear. In particular, note that the mass loss rate of specimen A is greater than that of specimen L. Among all the samples, A100 shows the steepest mass loss, followed by A50 and A20.

In the case of A series specimens, additional endothermic peaks are observed in the range of 100 °C to 200 °C, where CSH endothermic peaks appear. This indicates the decomposition of calcium acetate formed when acetic acid interacts with the cement paste [61]. In addition, at 600 °C to 800 °C, where the endothermic peak of CaCO_3_ is observed, calcium acetate is decomposed into CaO [61], resulting in an endothermic peak. This peak is substantial for A100 samples. For L samples, the heat peak of the DTA graph observed between 600 °C and 850 °C is due to the decomposition of both crystalline and amorphous CaCO_3_ formed by the interaction of citric acid and cement compositions [62,63,64].

Compared to the Ca(OH)_2_ peaks observed in the OPC graph, the Ca(OH)_2_ peaks in the A series specimens appear relatively smaller, particularly pronounced in the A100 specimen. This suggests a more definitive removal of OH^−^ ions in A100, with the Ca^2+^ ions and the resulting calcium acetate from Equation (5) being more readily identifiable in A100 compared to A50 and A20. In the L series specimens, the Ca(OH)_2_ peaks are similar to those in the OPC graph, but the CaCO_3_ peaks between the 600 °C to 800 °C range are sharper and more prominent in the L20 specimen compared to L100 and L50. This indicates that the product of Equation (4), resulting from the removal of OH^−^ ions and their reaction with citric acid, successfully points to the effective removal of OH^−^ ions in the L20 specimen.

### 3.5. SEM Analysis Results

**Figure 13:**
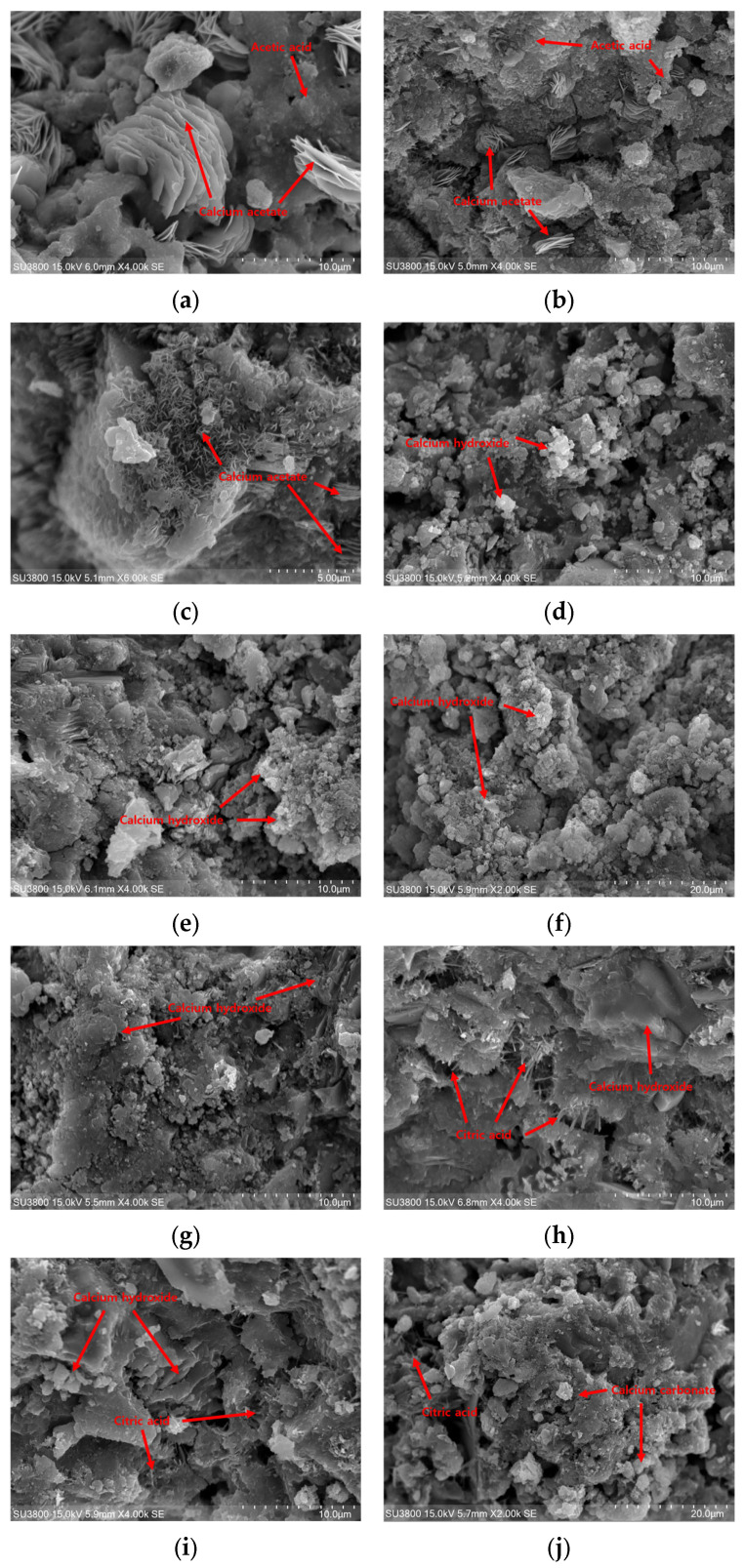

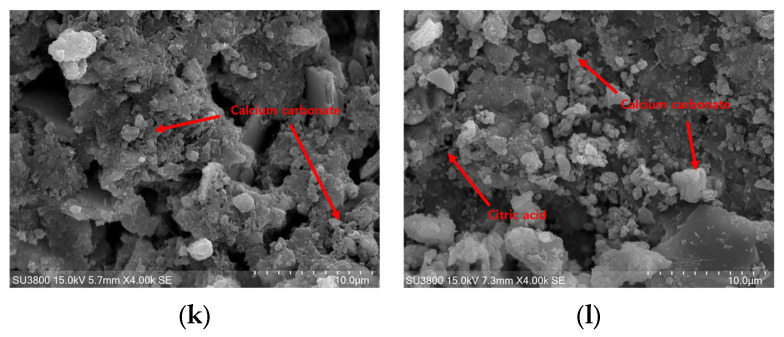
SEM analysis results: (**a**) A100−1; (**b**) A100−2; (**c**) A100−3; (**d**) A20−1; (**e**) A20−2; (**f**) A20−3; (**g**) L100−1; (**h**) L100−2; (**i**) L100−3; (**j**) L20−1; (**k**) L20−2; (**l**) L20−3.

Figure 13 shows the SEM analysis results of the A100, A20, L100, and L20 samples. In the A100 sample, the presence of acetic acid used for impregnation is confirmed [65], along with the formation of calcium acetate in a plate-like membrane form [64,65]. The reaction observed in Equation (5) indicates that acetic acid interacts with the cement hydration products, facilitating the removal of OH^−^ groups and forming stable calcium acetate compounds. The presence of these compounds signifies successful modification towards a P-type paste, as the removal of OH^−^ groups is important for this transformation. On the contrary, the A20 sample observed fewer plate-like calcium acetate structures, with a predominance of calcium hydration products resulting from the cement hydration reaction as described in Equation (5) [66,67]. These differences suggest that diluted solutions are less effective in promoting calcium acetate formation, indicating a dependent reactivity in which exposure to concentrations of relatively high proportions of acetic acid solutions enhances conversion to P-type properties.

In the L100 sample, unreacted CH is observed [66,67], which is a hydration product of cement that did not react with citric acid as in Equation (3). The presence of needle-shaped prism forms of citric acid [68] further corroborates the limited interaction between citric acid and CH in this sample. However, the observed structures suggest that citric acid begins to alter the cement matrix, albeit not as extensively as in other samples. The L20 sample, in contrast, shows a mixture of calcium carbonate [69,70] and the products of Equation (4), indicating a more balanced reaction process. The formation of calcium carbonate signifies successful neutralization and removal of OH^−^ groups by citric acid, which is crucial for the P-type conversion. The presence of both citric acid and calcium carbonate in distinct forms emphasizes effective interaction and reactivity within this sample.

Comparatively, the reactants in Equations (3)−(5) were significantly more pronounced in the A100 and L20 samples than in the A20 and L100 samples, respectively. The enhanced reactivity and well-defined products observed in A100 and L20 samples indicate a successful transformation towards P-type cement paste. The removal of OH^−^ groups and the formation of specific compounds, calcium acetate in A100 and calcium carbonate in L20, are critical indices of this success. Therefore, the SEM analysis results validate the experiment objective of producing P-type cement paste, demonstrating that extended impregnation concentration of solutions and specific acid interactions are pivotal in achieving this transformation.

### 3.6. EAA Analysis Results

**Figure 14:**
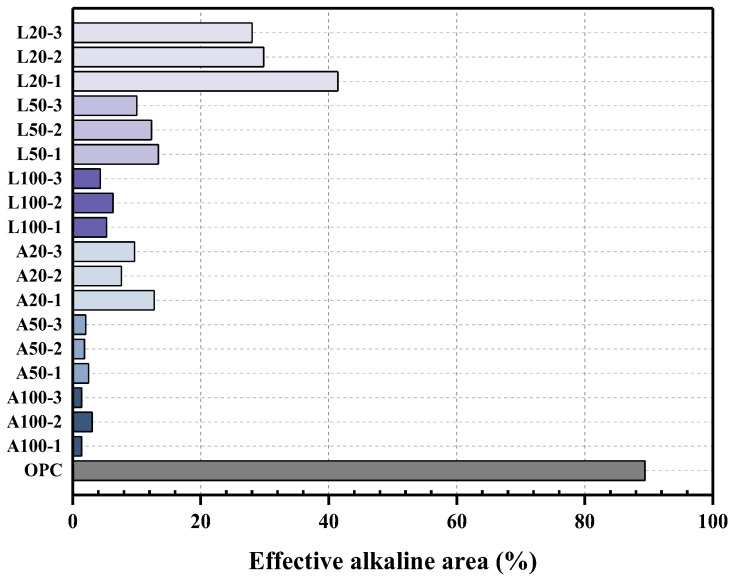
EAA test result.

The following Figure 14 presents the results of the EAA test, where the EAA was calculated using image processing. In the case of OPC, 89.41% of the total area reacted with phenolphthalein and was calculated as the effective region. For the A specimens, A100 exhibited the lowest effective region values, ranging from a minimum of 1.39% to a maximum of 3.04%. Meanwhile, A20 showed higher effective region values, ranging from a minimum of 7.62% to a maximum of 12.73%. Similarly, for the L specimens, L100 exhibited low effective region values, ranging from 4.31% to 6.3%, while L20 showed significantly higher values, ranging from 28.05% to 41.42%.

The notably high effective alkaline region observed in OPC is attributed to the strong alkalinity of the cement paste, which reacts with phenolphthalein to produce a purple coloration. In contrast, the A and L specimens were impregnated in an acidic solution, which reacted with the cement paste, removing OH^−^ groups and lowering the pH. As a result, phenolphthalein did not react, leading to a colorless response.

In particular, the lower effective alkaline region in the A specimens suggests that impregnation in acetic acid, which has a higher acidity, more effectively removed the internal OH^−^ groups compared to the L specimens, which were impregnated in lemon juice. This indicates a higher efficiency in producing P-type specimens.

### 3.7. Electrical Resistivity Results

**Figure 15:**
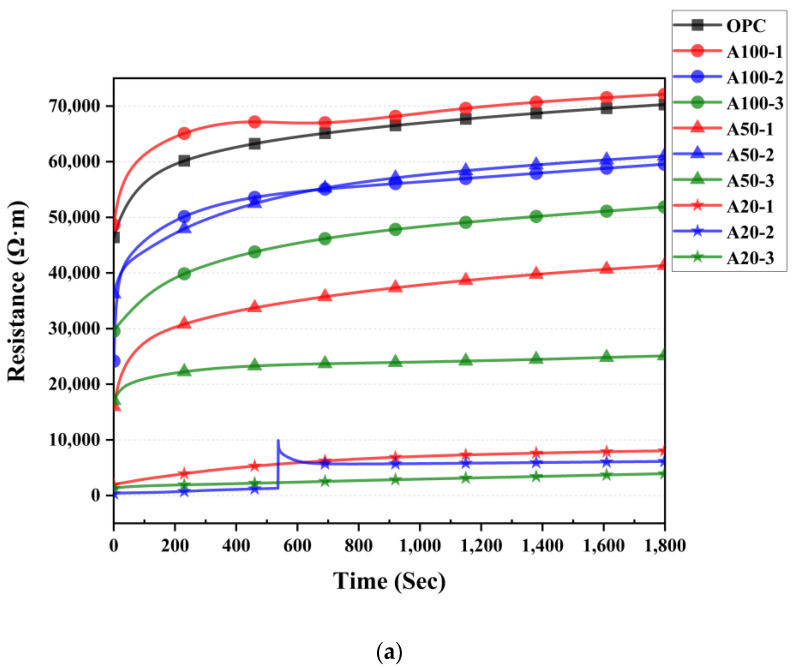

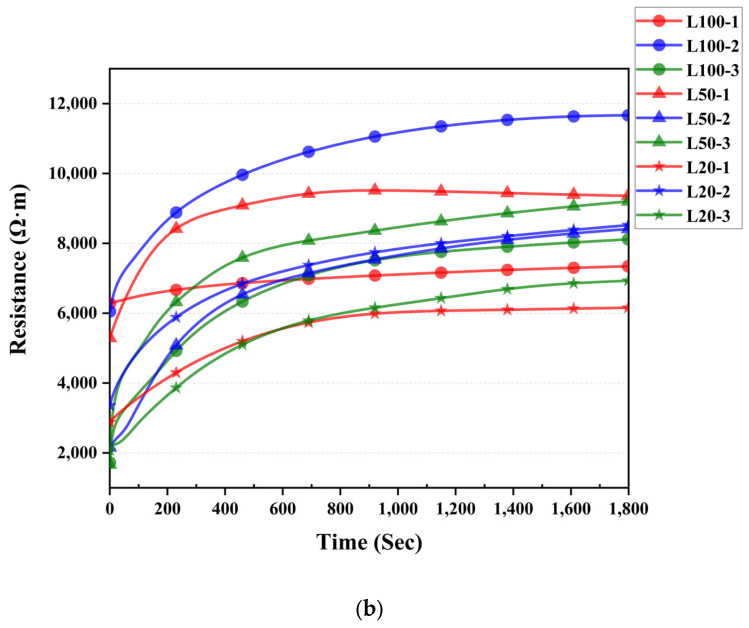
Electrical resistance test result: (**a**) OPC and A specimens; (**b**) L specimens.

Figure 15 presents the measured resistance values for each specimen. OPC, A100, and A50 specimens showed increasingly high resistance values. OPC and A100-1 have the highest resistance value, exceeding 70,000 Ω. The highest resistance values for the A100-2 and A50-2 are approximately over 60,000 Ω. The maximum resistance value was shown by approximately 50,000 Ω, 40,000 Ω, and 20,000 Ω on the A100-3, A50-1, and A50-3. The L100 and L50 then showed similar resistance values, while the A20 and L20 showed the lowest resistance values. Specifically, the maximum resistance values of L20-2 and A20-2 are approximately 8500 Ωand 9700 Ω, respectively. In the A20-2 case of Figure 15a, the radical peak in the 500–600 s range is a temporary peak and can be ignored.

When comparing the resistance values of Test A and Test L, it can be seen that the lower the dilution concentration of the acidic solution, the lower the resistance value. This is related to the amount of water used for the dilution of the solution, suggesting that the higher the amount of water contained, the lower the resistance value, and the study of Peng et al. [71] shows that the higher the W/C of the cement paste, the lower the electrical resistance value.

The resistance value of the test subject impregnated for 2 days tends to be higher in common with A and L test specimens. This is due to the removal of OH^−^ by an acidic solution in the process of Equations (3)−(5); calcium acetate and calcium carbonate are produced by removing the OH^−^ from the acidic solution, and these products can change the porosity of the cement matrix and affect the electron flow at a high density [72]. According to a study by Elkhaldi [72], the electrical resistance of the carbonized cement paste was measured, and the electrical resistance of the carbonized cement paste was higher than that of cement with full alkaline. In short, high alkalinity made a low electrical resistance. Therefore, the large resistance value of the 2-day impregnation test of A and L is considered to be due to the presence of products such as calcium acetate and calcium hydroxide generated after the OH^−^ of calcium hydroxide is removed, which is considered to have been successful in removing OH^−^ groups in the cement paste for the production of P-type, which is the purpose of this experiment.

### 3.8. Seebeck Coefficient Measurement Results

**Figure 16:**
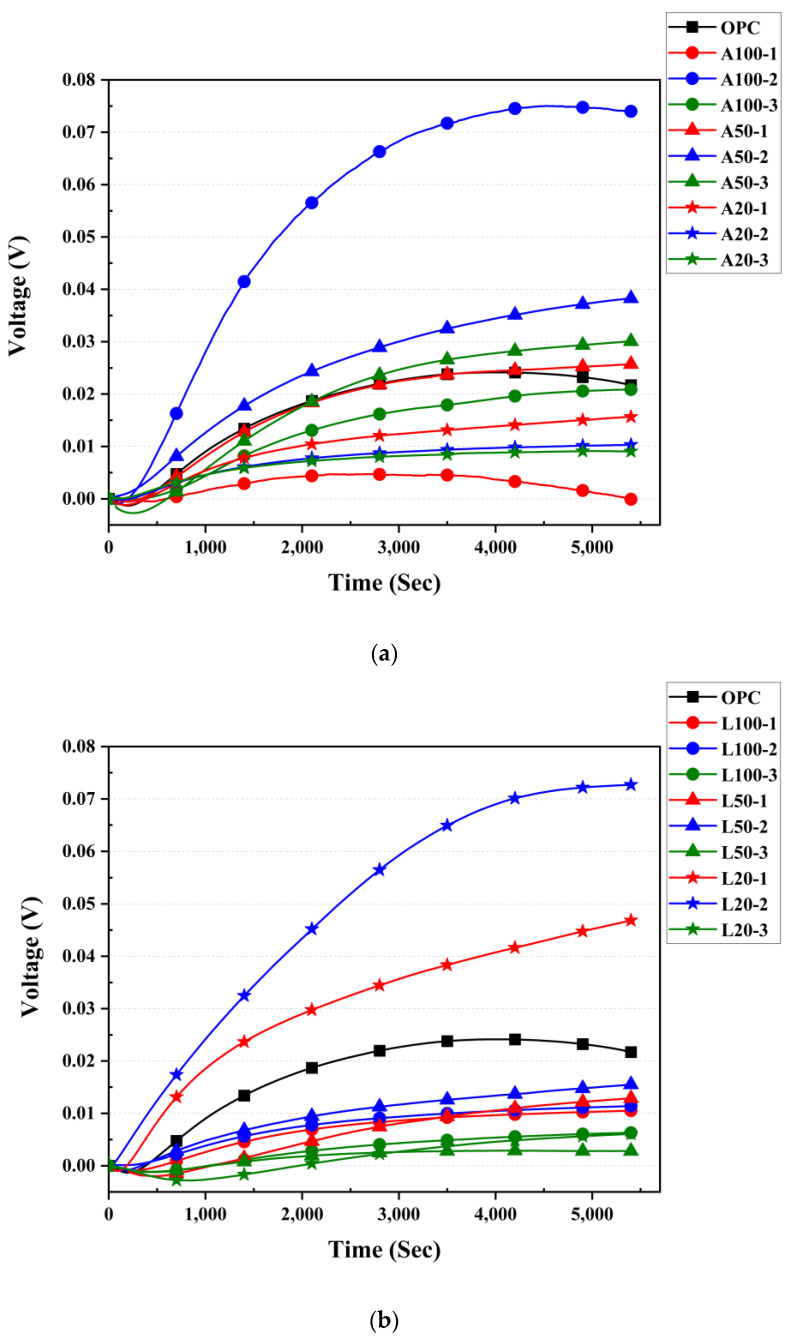
Voltage measurement values: (**a**) OPC and A specimens; (**b**) OPC and L specimens.

**Figure 17:**
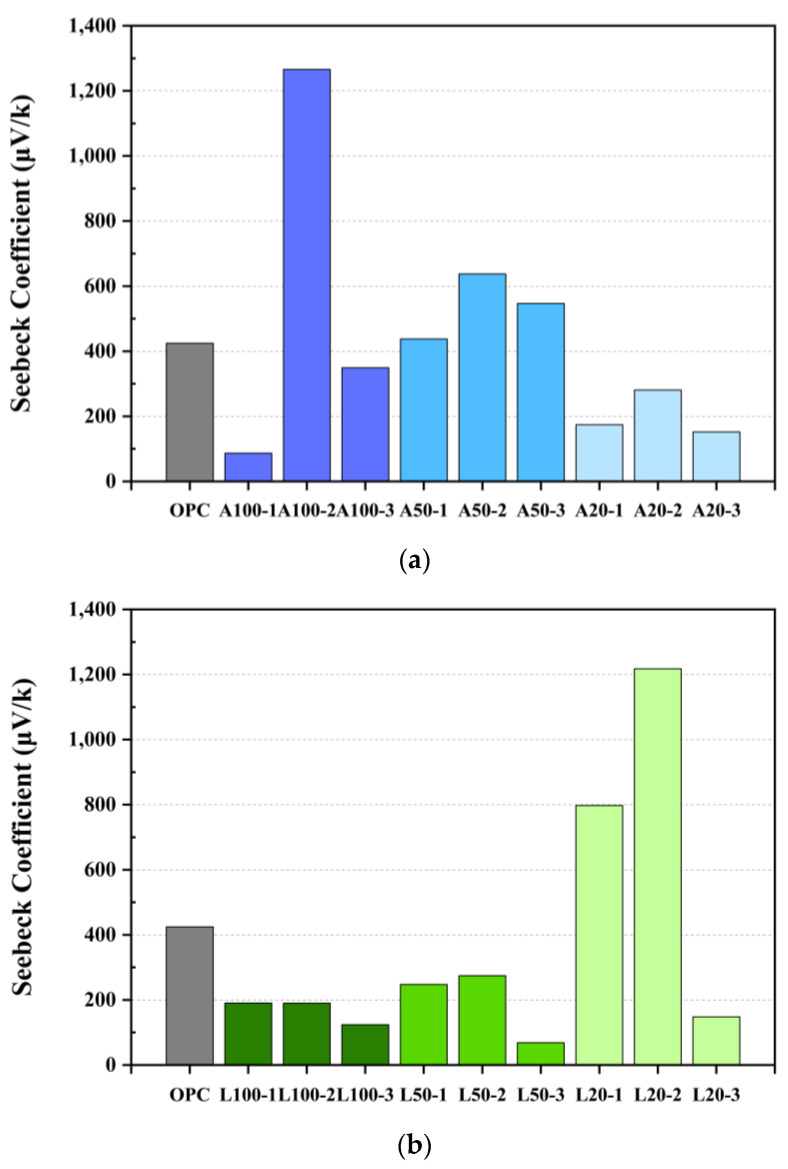
Seebeck coefficient result:(**a**) OPC and A specimens; (**b**) OPC and L specimens.

Figure 16 indicates the results of measuring the voltage generated by the sample using the thermoelectric effect. Contrary to the tendency of the electrical resistance value in Figure 16, the specimen on the 2 days where the resistance value was large showed a large current value. This is due to the Ca^2+^ ion generated during the reaction of Equations (3)–(5), and it is considered that the OH^−^ of the calcium hydroxide was removed at the same time as the product was formed, and the presence of the generated Ca^2+^ increased the current value. In the resistance value graph in Figure 16, it is deemed that on the 2 days, A and L tests produced the highest amount of calcium acetate and calcium carbonate, and at the same time, the Ca^2+^ ion was also generated at the maximum on the 2 days, indicating that the current value was the largest. This is due to the ion concentration phenomenon, as in the study of Toziunis [73], and is an important phenomenon for improving the SC performance of P-type cement paste with existing low SC values. These voltage measurements were then used to calculate the value of the final SC in Equation (2), as shown in Figure 17.

The SC for samples A100-2 and L20-2 were approximately 1270 µV/K and 1220 µV/K, respectively. These values are consistent with the goal of this study of effectively removing OH groups to produce P-type cement paste, resulting in a high SC. Subsequently, A series showed the SC in the order of A50 and A20, and sample L showed L50 and L100.

The highest SC observed in sample A100 is regarded due to the SEM analysis results in Figure 13. For the A100, calcium acetate, the product of Equation (5), was observed. In contrast, residual CH, which did not react with acetic acid, was present in the A20. This indicates that the specimen of A100 successfully removed the OH group from CH, resulting in Ca^2+^ ions. Through this, it can be explained that the specimen of A100-2 exhibits the highest SC value. After that, it can be confirmed that calcium acetate is generated by reacting with Ca^2+^ ions and acetic acids, and the ion concentration decreases, resulting in a decrease in the value of the SC in specimen A100-3.

In addition, the SEM analysis of the L20 sample observed calcium carbonate produced by the reaction of CH with citric acid, and the L100 contained unreacted citric acid and CH. This result is due to the response of Equation (4), which requires water as a reactant to form calcium carbonate. As a result, it is considered that a significant amount of calcium carbonate was observed in the L20 specimen, which was diluted with water and packed using the acidic solution used. Therefore, Ca^2+^ for producing the products of Equations (3) and (4) was best observed in the L20 specimen, which is regarded as an indicator that successfully removed the OH^−^ of calcium hydroxide. At this time, the specimen containing the most Ca^2+^ ions was L20-2, and then Ca^2+^ reacted with the citric acid to produce calcium carbonate, resulting in a decrease in the value of SC.

The highest SC in the same sample group was found in both A and L samples. This result is attributed to the optimum period of OH group removal and Ca ionization during acid solution impregnation in cement compositions. According to Bertron et al. [74], in the study, cement paste was impregnated with organic acid to observe the change in Ca^2+^ ion concentration of the paste according to the impregnation time. As a result of comparing the impregnation times of 24, 48, and 72 h used in the study of Bertron et al. [74], it was observed that the cation concentration was the highest at 48 h, followed by 72 h and 24 h. This explains why samples impregnated for 48 h showed consistently high SC in this experiment. It is also believed that the phenomenon of SC showing the highest value in the test specimen on day 2 in the L test is also due to the effect of the chain effect of CH. In the study of Kasselouri-Rigopoulou [75], it was confirmed that not all of the citric acids reacted with CH, but the products of Equations (3)–(5) reacted with the citric acid again to form carbon dioxide, water, and citric acid. In this way, the chain effect in which the products and reactants are exchanged and the reaction occurs in a chain may occur in the test specimen of L. However, this reaction is predicted due to the low carbon dioxide content in the air at room temperature [29]; ion generation until the complete consumption of the citric acid requires a considerable reaction time and is expected to be active. Therefore, Ca^2+^ ion concentration is expected in the test specimen of L20-2, and a high SC value is excluded. In fact, the fabrication of p-type cement paste requires a fairly complex composition process and shows a low value of SC. However, as in the study of Tzounis [73], the reason for showing a relatively high value of SC of the P-type test in this study is considered to be a disproving of the ion concentration [75] described above.

## 4. Conclusions

In this study, in order to produce a P-type cement paste by removing OH^−^ groups in the cement paste, acetic acid and pure lemon juice were vacuum impregnated for 24 h, 48 h, and 72 h at three concentrations: 100%, 50%, and 20%. After that, in order to evaluate the impregnation conditions with the optimum electricity production capacity, the chemical and mechanical properties of each test were evaluated, and the SC was calculated. The results of this experiment are as follows.

1. Both the concrete impregnated in the acid and the lemon juice solution showed high SC values of the specimens impregnated for 48 h within the solution ratio. This is due to the ability of the cement paste to generate Ca^2+^ ions according to the acidic solution impregnation time, and the values were large in the order of 48 h, 72 h, and 24 h.
2. In the case of the specimen impregnated with acetic acid, the largest value of the SC was A100-2 at 1270 μV/K, and in the case of the specimen impregnated with lemon juice, the SC value was 1220 μV/K, which was the largest in L20-2. This is due to Equations (3)–(5), and it can be seen from the SEM results in Figure 13 that a large number of products produced after OH-group removal are observed in A100 and L20, respectively.
3. The porosity analysis demonstrated a clear correlation between porosity and compressive strength in acid-impregnated cement paste. Higher porosity generally led to reduced compressive strength due to decreased gel-to-pore space ratio resulting from acid-induced dissolution of CH and demineralization of CSH. Specifically, acetic acid impregnation significantly increased porosity and lowered strength due to its higher acidity. Conversely, citric acid-impregnated L specimens exhibited improved compressive strength, attributed to the formation of calcium carbonate films, which enhanced surface densification and effectively sealed internal pores. These observations clarify the strength differences between the A and L specimens.
4. The specimens impregnated in acetic acid demonstrated a low effective alkaline region, indicating effective OH^−^ removal, while the high SC value suggests enhanced efficiency as a P-type specimen. However, the high porosity and consequently low compressive strength indicate poor usability of the acetic acid-impregnated specimens, ultimately making them unsuitable for use as P-type cement paste.

Through this study, it was confirmed that the optimal impregnation environment for manufacturing P-type cement paste was L20-2, which was impregnated for two days in a solution diluted with 20% lemon juice. After this study, it is necessary to conduct research on the manufacture of electrically produced cement composites through the Peltier effect using P-type and N-type cement composites.

## Figures and Tables

**Table 1:** Properties of OPC [12].

Chemical Composition (%)						Physical Properties	
SiO_2_	Al_2_O_3_	Fe_2_O_3_	CaO	MgO	SO_3_	Specific gravity (ton/m^3^)	Fineness (cm^2^/g)
21.1	6.2	3.2	62	3.3	22	3.2	3200

**Table 2:** Properties of acetic acid (CH_3_COOH).

Physical Properties at 20 °C			
Freezing Point, °C	Boiling Point, °C	Vapor Pressure, kPa	Density, kg/m^3^
16.64	117.87	15.7	1049.55

**Table 3:** Chemical properties of acetic acid (CH_3_COOH) and lemon liquid.

Components (µg/cm^2^)	Acetic Acid	Lemon Liquid
Mg	-	1.336
Al	0.1069	0.1968
Si	0.1005	0.3064
P	-	1.439
S	-	1.2823
Cl	0.3321	0.7999
K	-	20.416
Ca	-	1.1595
Fe	0.243	0.3708
C_6_H_10_O_5_	10,185.13	10,158.31

**Table 4:** Capacity of each acidic solution mix design.

Components	Acetic Acid (L)	Lemon Liquid (L)	Water (L)
OPC	-	-	-
A100	4	0	0
A50	2.25	0	2.25
A20	0.9	0	3.6
L100	0	4.5	0
L50	0	2.25	2.25
L20	0	0.9	3.6

## Data Availability

The original contributions presented in this study are included in the article. Further inquiries can be directed to the corresponding author.
